# Egg Laying Decisions in *Drosophila* Are Consistent with Foraging Costs of Larval Progeny

**DOI:** 10.1371/journal.pone.0037910

**Published:** 2012-05-31

**Authors:** Nicholas U. Schwartz, Lixian Zhong, Andrew Bellemer, W. Daniel Tracey

**Affiliations:** 1 Neuroscience Program, Trinity College of Arts & Sciences, Duke University, Durham, North Carolina, United States of America; 2 Pharmacology Science Training Program, Duke University, Durham, North Carolina, United States of America; 3 Department of Neurobiology, Duke University, Durham, North Carolina, United States of America; 4 Department of Cell Biology, Duke University, Durham, North Carolina, United States of America; 5 Department of Anesthesiology, Duke University Medical Center, Durham, North Carolina, United States of America; AgroParisTech, France

## Abstract

Decision-making is defined as selection amongst options based on their utility, in a flexible and context-dependent manner. Oviposition site selection by the female fly, *Drosophila melanogaster*, has been suggested to be a simple and genetically tractable model for understanding the biological mechanisms that implement decisions [1]. Paradoxically, female *Drosophila* have been found to avoid oviposition on sugar which contrasts with known *Drosophila* feeding preferences [1]. Here we demonstrate that female *Drosophila* prefer egg laying on sugar, but this preference is sensitive to the size of the egg laying substrate. With larger experimental substrates, females preferred to lay eggs directly on sugar containing media over other (plain, bitter or salty) media. This was in contrast to smaller substrates with closely spaced choices where females preferred non-sweetened media. We show that in small egg laying chambers newly hatched first instar larvae are able to migrate along a diffusion gradient to the sugar side. In contrast, in contexts where females preferred egg laying directly on sugar, larvae were unable to migrate to find the sucrose if released on the sugar free side of the chamber. Thus, where larval foraging costs are high, female *Drosophila* choose to lay their eggs directly upon the nutritious sugar substrate. Our results offer a powerful model for female decision-making.

## Introduction

Oviposition site selection by the *Drosophila melanogaster* female has been suggested to be a simple model for the study of decision-making processes [1], [2]. Indeed, the powerful genetic tools available for neural and molecular circuit mapping in *Drosophila* make this an attractive idea. For a biologically meaningful model of decision making, neural circuits should produce outcomes that result in a selective advantage to the organism. Indeed, errors made in oviposition site-selection would impose a significant energetic cost (and selective disadvantage) through the resources wasted in oogenesis. In contrast, natural selection would favor the evolution of neural circuits that generate oviposition preferences for sites favoring survival of offspring.

Given these assumptions, it was surprising when results from a recent study showed that female *Drosophila melanogaster* actively avoid laying eggs on a medium containing sucrose if given the choice of alternate medium lacking the sucrose [1]. When given the choice between a sucrose-containing medium and a plain medium, the flies were found to prefer –laying eggs on plain medium [1]. Even more surprisingly, when given the choice between a sucrose containing medium and a bitter or salty medium the flies still avoided the sugar [1]. We wondered how egg-laying choices would be selectively advantageous, given the preference of a non-nutritive substrate in each of these examples. We thus sought to further investigate these findings.

## Results and Discussion

**Figure 1:**
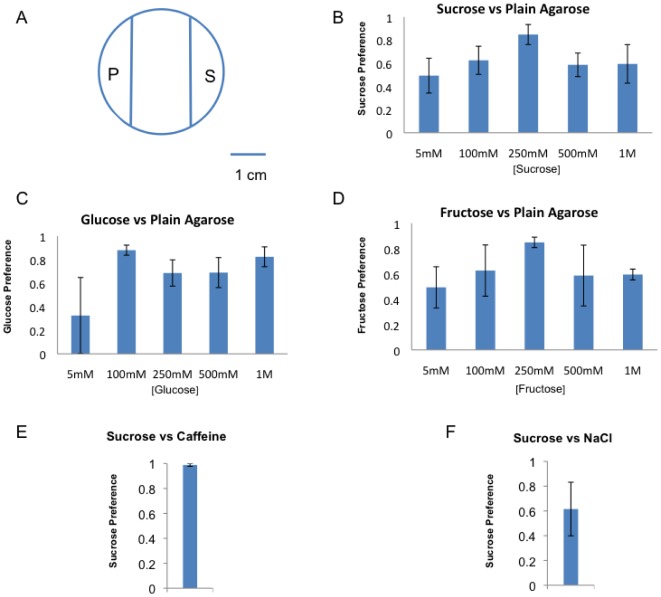
*Drosophila* show a clear preference for laying eggs on sugar containing substrates. (A) Schematic representation of the egg laying chamber showing the relative position of both substrates. The two 1% agarose substrates (P indicates plain, S indicates Sugar) were separated by 3% agarose middle zone. (B) Flies showed a preference for sucrose over plain agarose for egg laying at most concentration tested. (5 mM (n = 5, p<0.05, average total number of eggs = 33), 100 mM (n = 13, p<0.001, average total number of eggs = 65), 250 mM (n = 5, p<0.001, average total number of eggs = 73), 500 mM (n = 10, p<0.001, average total number of eggs = 56), and 1000 mM (n = 5, p<0.05, average total number of eggs = 50)) of sucrose. (C) Glucose was also preferred to a plain agarose substrate (5 mM (n = 4, p>0.05, average total number of eggs = 37), 100 mM (n = 10, p<0.001, average total number of eggs = 56), 250 mM (n = 5, p<0.01, average total number of eggs = 105), 500 mM (n = 5, p<0.01, average total number of eggs = 66), and 1000 mM (n = 5, p<0.001, average total number of eggs = 89)) (D) Flies showed a preference for fructose over plain at each concentration tested. (5 mM (n = 5, p<0.05, average total number of eggs = 28), 100 mM (n = 5, p<0.05, average total number of eggs = 45), 250 mM (n = 3, p<0.01, average total number of eggs = 43), 500 mM (n = 6, p<0.05, average total number of eggs = 42), and 1000 mM (n = 5 m, p<0.001, average total number of eggs = 48)) (E) Flies showed a nearly absolute preference for sucrose (100 mM) over caffeine (10 mM) as an egg laying substrate (n = 4, p<0.001, average total number of eggs = 83). 1f) Flies showed strong preference for sucrose (100 mM) over NaCl (100 mM) as an egg laying substrate(n = 5, p<0.05, average total number of eggs = 39). Student t-tests were performed for statistical analysis with one-tail p value.

We first gave the wild type Canton-S flies the option to lay eggs on a sucrose-containing agarose medium versus a plain agarose medium (Figure 1A). Unexpectedly, we observed a clear preference for oviposition on sucrose at each of the five concentrations examined (5 mM, 100 mM, 250 mM, 500 mM and 1 M) (Figure 1B). In addition, flies also showed a similar preference to lay eggs on agarose media sweetened with glucose or fructose versus the plain agarose medium (Figure 1C, 1D). Combined, these results indicated that female *Drosophila* of the Canton-S genotype show a clear preference for oviposition upon sugar-containing substrates over a non-sugar substrate under these experimental conditions.

We also found that flies given the choice of egg laying on sucrose (100 mM) and the bitter compound caffeine (10 mM) showed a nearly complete preference for the sucrose (Figure 1E). Similarly, flies given the choice between sucrose (100 mM) medium and NaCl (100 mM) showed a preference for the sugar over the salt (Figure 1F). Again, these results were unexpected considering a previous study which found that flies showed a strong preference for laying eggs on the bitter and salty medium over a sucrose medium [1].

Nevertheless, our results are consistent with the known *Drosophila* feeding preference for sugary foods and avoidance of feeding on bitter or salty foods [3]–[7]. Thus, it seems likely that the oviposition preference on sugars that we observed reflects a normal innate behavioral program for *Drosophila* egg laying.

Given the contrast with the earlier published results, we sought to find differences in the experimental approaches that might explain the observed female behavior. We noted that the physical dimensions of our egg laying chambers differed from those in the prior study. Our egg-laying chambers consisted of Petri dishes 35 mm in diameter (an area of 964 mm^2^, Figure 1A). Petri dishes of this size are commonly used in *Drosophila* laboratories for embryo collections. Indeed, it is common practice for *Drosophila* researchers to perform embryo collections on media that contain sucrose. In contrast, the prior study that found avoidance of sucrose used chambers that were quite small in comparison (14.5×18.4 mm, or 266 mm^2^) [1].

**Figure 2:**
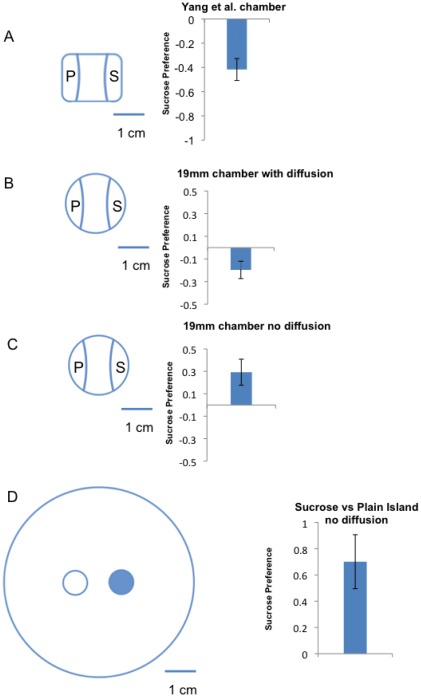
Substrate size and diffusion gradients affect *Drosophila* egg laying choices. In each panel the egg laying chamber is schematically shown on the left and the oviposition preference observed for the indicated chamber is shown on the right. (A) Flies preferred plain over sucrose substrate for egg-laying in the chambers that were used in Yang et al. (n = 3, p<0.05, average total number of eggs = 67) (B) Flies also preferred plain over sucrose substrate for egg-laying in small cylindrical chambers (n = 22, p<0.01, average total number of eggs = 58). (C) Flies preferred sucrose over plain substrate for egg-laying in small cylindrical chambers in an experimental setup without diffusion (n = 11, p<0.05, average total number of eggs = 76) (D) The flies preferred sucrose over plain substrates for egg-laying in chambers in which sucrose and plain islands were spaced 5 mm apart when there was a barrier to diffusion(n = 13, p<0.001, average total number of eggs = 25). Student t-tests were performed for statistical analysis with one-tail p value.

To test whether the use of small chambers might result in an egg-laying environment that would alter the female preference for oviposition on sucrose we performed egg laying experiments in small rectangular egg-laying chambers that were identical to those used in the prior study (Figure 2A). Our results were consistent with those of the prior report, as we observed a mild preference for oviposition on plain agarose relative to sucrose (Figure 2A). In addition, we examined very small cylindrical chambers (19 mm diameter) that had a similar surface area (283 mm^2^) (Figure 2B). Again, results using these chambers indicated a mild preference for the plain medium over the sucrose-containing medium (Figure 2B). Clearly, the probability of egg-laying on the plain agarose medium was increased in egg laying assays using small chambers. These results suggested the possibility that the valence of sucrose can be switched from being very attractive to being mildly unattractive depending on the size of the habitat that is available to the female flies while making the oviposition choices.

Indeed, it is known that confinement of *Drosophila* to small spaces can dramatically affect the normal behavioral programs. For example, wing expansion of newly eclosed flies is strongly inhibited by confinement [8] which shows that *Drosophila* have an innate capacity to measure the space available in their surrounding environment [8]. Furthermore, this measurement of space is clearly used by *Drosophila* in neural circuits that produce behavioral outcomes [8]. Thus, it seemed possible that a measurement of space is used by female flies when making oviposition choices. This idea was suggested previously by Yang et al. in order to explain reduced avoidance of sucrose in contexts where the egg laying choices were separated by increased distances [1].

However, in the assays of Yang et al., as well as in our assays as performed thus far, the chamber size always co-varied with the distance between the alternative egg laying substrates [2]. In the large chambers the alternate food choices are indeed farther apart. But, there is a much greater surface area and volume available to the female flies during the course of the experiment. We thus tested whether it was the overall size of the larger chambers, or the distance between the substrates in the larger chambers, that was the critical variable that caused the switch of preference between sucrose and plain agarose substrates.

To achieve this we allowed the flies to choose between sucrose-containing agarose and plain agarose substrates that were close together, but in the context of the larger chambers. We prepared chambers with two egg-laying islands (diameter 7.5 mm) that were separated by a distance of 5 mm. One island contained 100 mm sucrose 1% agarose and the other island contained plain 1% agarose, the two islands were surrounded by an unfavorable 3% agarose substrate. If distance between the egg laying substrates affects the decision of female flies to be attracted to sucrose, then the sucrose should still be avoided in this context. This was not observed. The results showed a clear preference for oviposition on sucrose (Figure 2D). This indicates that chamber size, and not the distance between the substrates *per se*, regulates preference for sucrose as an egg laying substrate.

**Figure 3:**
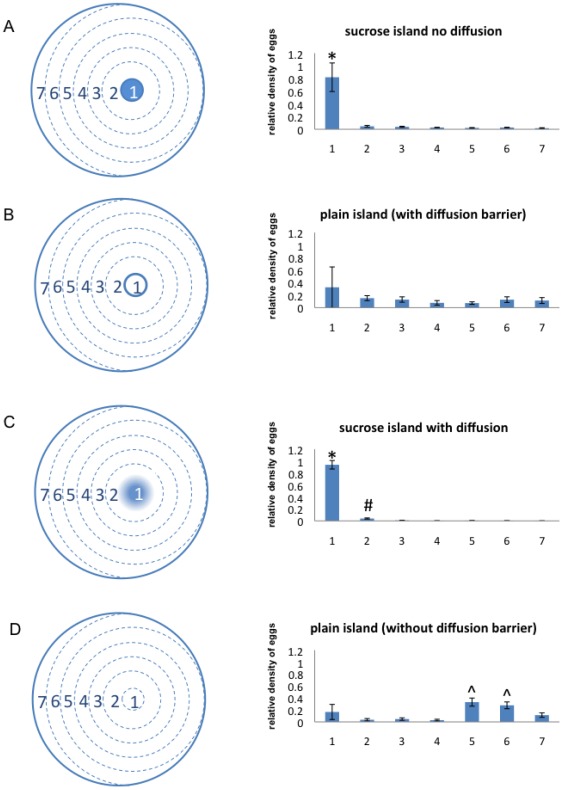
In large chambers, *Drosophila* prefer egg laying on sugar even when given the option to lay eggs close to the sugar or with diffusion gradients. In these single island experiments, flies had the options of laying eggs at any location on the substrate. For the purpose of quantification, the petri dish was divided into 7 zones. Relative densities of eggs were calculated for each zone. (A) With 100 mM sucrose island experiments and no diffusion, females laid a significantly greater fraction of eggs in the sucrose island than in any other zone (as determined by a one-way ANOVA and Tukey's Multiple Comparison Test; * indicates p<0.05 as compared to each other zone) (n = 13, average total number of eggs = 24). (B) The island itself was only mildly attractive when filled with plain agarose (no significant differences by one-way ANOVA and Tukey's Multiple Comparison Test) (n = 5, average total number of eggs = 37). (C) In a 100 mM sucrose island with diffusion, females strongly preferred the island as determined by a one-way ANOVA and Tukey's Multiple Comparison Test; * indicates p<0.05 as compared to each other zone; # indicates p<0.05 as compared to zone 3–7) (n = 17, average total number of eggs = 41). (D) A plain island was not attractive in comparison to the sucrose island used in C (^ indicates p<0.05 as compared to zones 1–4, and 7) (n = 8, average total number of eggs = 15).

To further investigate the effect of distance on the decision-making paradigm we designed an experiment that gave females the option of laying eggs over a range of distances but still within the context of the larger chambers. In this experiment, the small sucrose-containing agarose island was surrounded by a suitable plain 1% agarose egg laying substrate divided into six zones for the purposes of quantification (Figure 3). This scenario allowed the females to choose between laying their eggs directly upon the sucrose, or at any other possible distance from the sucrose within the chamber. Even under these circumstances, the females showed a very strong preference for laying eggs directly upon the sucrose island, as significantly more eggs were laid on the sucrose island than in any of the surrounding zones (Figure 3A). In contrast, there was no significant preference for laying eggs on the island in control experiments where the island contained plain 1% agarose (Figure 3B). A two-way analysis of variance (ANOVA) showed a trend towards a significant interaction (p = 0.0514) between island composition (sucrose versus no sucrose) and egg-laying zone, suggesting that the presence or absence of a sucrose island influences the distribution of egg laying across the plates. These results demonstrate that females prefer to lay eggs directly upon the sucrose even if given the option to lay eggs very close to the sucrose.

**Figure 4:**
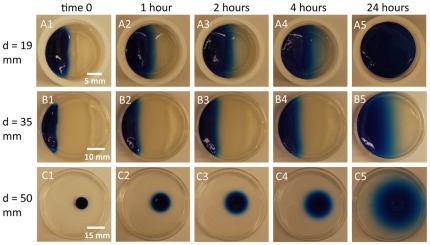
Visualization of diffusion in different experimental settings. The three different sized chambers (19 mm, 35 mm and 50 mm) were set up as described in the methods except that the 100 mM sucrose was substituted with 1% agarose with 1% ethanol, 0.025% w/v Bromophenol Blue and 0.025% Xylene Cyanol. The molecular weights of these dyes are approximately twice that of sucrose so these dyes will underestimate the diffusion of sucrose. The photos were taken at time points 0, 1, 2, 4 and 24 hours.

How can the preferences of females in our experiments be explained in biologically meaningful terms? One possibility is that in small chambers, laying eggs on the plain agarose is not strongly disadvantageous. This is likely to be the case because in small chambers, diffusion is expected to minimize the difference in sucrose concentration across the substrate in the time period required for embryonic development. For example, since the eggs laid upon the plain food will not hatch until 24 hours after egg laying the relevant sucrose concentration occurs at this later time point. Upon hatching in the small chambers, larvae are expected to easily locate the sucrose present in the chamber using well-described foraging behaviors [9]–[11]. In the small chamber setup, extensive diffusion occurs (Figure 4). This diffusion minimizes the difference in sucrose concentration at the time of larval hatching, reducing the cost of choosing to lay eggs on plain agarose, and could possibly by used by larvae to find the higher sucrose concentration on the opposite side of the chamber. These combined effects are expected to minimize any selective disadvantage to laying eggs on the plain side of the chamber.

In contrast, locating the sucrose containing food in the larger chambers increases the costs of the foraging required for the larvae to find the sucrose if they hatch on the plain agarose. Using a random foraging strategy, the difficulty of the foraging task is not a simple function of distance between the food choices. Rather, as described in Charnov's Marginal Value Theorem, the cost of foraging is a function of the distance between patches relative to the total possible search area [12]. The costs of foraging increase as search area increases, because in the context of a larger search area resource patches are more difficult to find. Indeed, this idea is consistent with our finding that the sucrose substrate is still preferred to the plain substrate, even when the female flies were given the option to lay eggs very close to the plain substrate in the larger chambers.

We utilized an impermeable plastic barrier to prevent the formation of a sucrose gradient in the island experiments (Figure 3A, 3B). This would require that hatched larval progeny find the sucrose containing food using a random search strategy. If female neuronal circuits actually evolved to evaluate environmental factors that influence larval ability to find the sucrose, then the presence of a diffusion gradient might influence the preference for egg laying on sucrose. This is because a diffusion gradient could theoretically be used by the larvae to locate the sucrose containing substrate.

To test whether sucrose diffusion could explain the altered egg laying preference in the small environment, we examined egg-laying preferences in small chambers in which diffusion of sucrose was not allowed. In this experiment, we prepared the chambers leaving a small gap between the sucrose containing substrate and the plain agarose substrate. This contrasts with the experiments above in which the two egg laying choices were connected by a 3% agarose layer following the method of Yang et al. [1]. Consistent with the hypothesis that diffusion would affect the behavioral outcome of the assay, we found that females actually preferred to lay eggs on sucrose in the small chambers when a diffusion gradient was not allowed to form (Figure 2C).

We also examined whether the presence of a diffusion gradient in the larger chambers would affect the female decision making process. In this experiment, we prepared a small 1% agarose sucrose island surrounded by a plain 1% agarose substrate but in the absence of the diffusion barrier (Figure 3C). This situation was similar to the single island experiment shown in Figure 3A since the females could choose to either lay eggs directly upon the sucrose island, or they could lay eggs at any other available location within the chamber. Unlike the situation in small chambers, the presence of the sucrose gradient did not significantly reduce the preference for the sucrose island in the context of the large chambers (Figure 3C, 3D). In experiments using a sucrose island, significantly more eggs were laid on the island and in Zone 2 than in the other zones (Figure 3C). Whereas in the presence of a plain island, there was no preference for laying eggs on or near the island (Figure 3D). Interestingly, significantly more eggs were laid near the edges of the dish in this condition (Figure 3D), suggesting a possible preference for edges in the absence of gustatory stimuli (as in Figure 3A, 3C). In Figure 3B, the hard plastic diffusion barrier apparently cancels out this effect, presumably due to the creation of a competing edge by the diffusion barrier itself. A two-way analysis of variance (ANOVA) showed a significant interaction (p<0.0001) between island composition (sucrose versus no sucrose) and egg-laying zone when comparing the conditions of Figure 3C and 3D. These results are consistent with the idea that the foraging task is more difficult in the context of a large search area and therefore there is a selective advantage to egg-laying directly upon the sucrose even when a gradient is present.

To provide an estimate of the extent of diffusion in our various experimental contexts, we added dye to the egg laying substrates and visualized the movement of the dye over time (Figure 4). The dye movement in the first four hours of the experiment was most relevant since the great majority of eggs in our experiments were deposited in this time interval (NS, LZ, WDT unpublished observations). This peak of egg-laying occurred shortly after lights off in the environmental chamber and coincided with the previously described circadian peak of egg laying that occurs shortly after dusk [13].

The diffusion of the dye that was observed in the small egg laying chambers had reached the opposite side of the chamber even at these early time points. Importantly, this contrasted with larger chambers in which a significant proportion of the chamber surface remained free of dye. Thus, similar behavior of sucrose would make the plain agar a discrete choice in comparison to the sucrose agar in larger chambers. In contrast, the plain agar in small chambers would actually contain sucrose at the time that the females were making their egg laying decisions.

**Figure 5:**
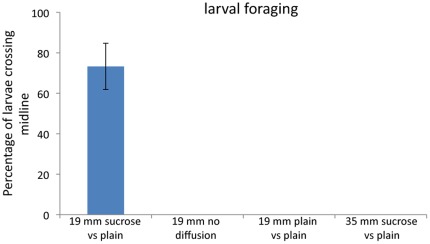
*Drosophila* larvae crawl towards a sucrose source in the presence of a diffusion gradient. In these experiments larvae were placed in 19 mm chambers containing plain and 100 mM sucrose egg-laying substrate. In chambers where diffusion was not allowed, no larvae placed on the plain substrate crossed over to the sucrose substrate within 30 minutes of observation (n = 15). In chambers where diffusion was allowed, 73% (SEM = 11.42%) of larvae crossed from the plain substrate to the sucrose substrate during the 30-minute observation period (n = 15). In chambers where both halves contained plain substrate (a control for the diffusion chamber) no larvae crossed the midline within 30 minutes (n = 15).

The key unanswered question in our experiments is whether or not the larvae that hatch on the plain food in the different environments are able to find the sucrose containing food. If newly hatched larvae are easily able to find the sucrose, then there is little cost to female avoidance of sucrose during oviposition. Thus, we measured the amount of time it took for larva to transverse from the plain agar substrate to the sucrose substrate under the different experimental conditions used (Figure 5). We placed three newly hatched Canton-S larvae on the far edge of the plain agarose in the experimental chambers. We then observed the larvae for a period of thirty minutes and measured the time until the larvae crossed to the opposite side of the chamber. Five trials, each with three larvae, were performed for four experimental conditions: small (19 mm) cylindrical chambers with sucrose and diffusion, small (19 mm) cylindrical chambers without diffusion, larger (35 mm) cylindrical chambers with diffusion, and small (19 mm) cylindrical chambers with plain agarose on both sides.

In 19 mm cylindrical chambers with diffusion, 73% (SE = 11.42%) of larva crossed from the plain side to the 100 mM sucrose side of the chamber within the 30 minute observation period (Figure 5). At least two out of the three larvae crossed into the sucrose side in all five of the trials. For these larvae, the amount of time needed to migrate into the sucrose agarose ranged from one to 25 minutes with an average crossing time of 768±140 seconds. In these experimental chambers, larval movement towards the opposite side depended on the presence of a sucrose gradient, because larvae never crossed into the sucrose when diffusion was not allowed (Figure 5). In control experiments with plain agarose on both sides of the chamber, larva never crossed the midline into the opposite side (Figure 5). In contrast to the results in 19 mm chambers, no larvae migrated from plain agarose to the sucrose agarose in 35 mm side even in the presence of a diffusion gradient (Figure 5). Thus, the diffusion-dependent crossings seen in the small chambers were clearly distinct from the absence of crossings seen in all other experimental conditions (two-tailed Fisher's Exact Test p<0.0001). Combined, these data demonstrate that newly hatched larvae are indeed able to follow diffusion gradients from the plain to the sucrose side in the small chambers. This larval foraging capability demonstrates a reduced cost for egg laying away from sucrose in these conditions.

Our results expand on those of recent studies, which suggested that the egg laying decisions of female *Drosophila* are context dependent [1], [2], [14]. However, our results differ from prior studies in several important ways. First, we find that there is an innate attraction to sugars including sucrose as egg-laying substrates. This was not previously observed. Importantly, this means that sugars are not innately aversive to female flies. We uncovered this innate attraction through the use of egg-laying environments that were larger than the previously used environments. We propose that this larger environment increases the costs of larval foraging and the egg-laying circuitry is equipped to deal with this potential cost. Second, we find that although females do show an apparent avoidance of sucrose in some experimental contexts, this only occurs under conditions where diffusion minimizes the difference in sucrose concentration at the time of larval hatching. In addition, the larvae are able to utilize the sucrose gradient and migrate to the higher sucrose in the small chambers. Because of this, the reproductive cost of making either decision at the time of egg laying is essentially equal.

We propose that female egg laying circuits have co-evolved with larval life history strategies and that they are consistent with expected larval foraging costs. When larval foraging costs are high, the female flies show a preference for laying eggs directly upon the more nutritious substrate. A still unexplained finding is the observation that female flies do show a mild but consistent preference for the alternate food in the small chambers when diffusion is present. An interesting unanswered question is the selective advantage of sucrose avoidance in these circumstances.

It is important to note that the conditions in small chambers are unlikely to resemble egg-laying conditions that would be encountered in the wild. Indeed, it is unlikely that female flies in the wild would ever choose to lay eggs on food sources as minute as the substrates that are available in the small 19 mm chambers.

We can only speculate why avoiding sucrose might be an “optimal” strategy under the extremely limited circumstances in which we find it to occur. Perhaps in small chambers females detect the gradual increase of sucrose concentration that occurs on the plain side of the chamber (due to diffusion). The females might also be capable of detecting the decrease in sucrose concentration on the opposite side of the chamber that occurs over time. If female flies are capable of temporal integration of sucrose measurements, it would be advantageous to choose the substrate on the side of the chamber where sucrose is perceived as increasing (and to avoid the decreasing side).

## Methods

### Fly Husbandry

*Drosophila melanogaster* of the Canton-S strain were maintained on a cornmeal molasses fly food medium at 25°C, 75% relative humidity, on a 12 hour light-dark cycle (lights off at 8:00P.M.). For all egg laying assays freshly collected virgin female flies were held in the absence of males for a period of 4 days. Following this four-day period, mating was allowed to proceed in vials containing cornmeal molasses medium. The mating period was for two hours with 5 virgin male and 5 virgin female *Drosophila* in each vial. Direct observation indicated that this procedure allowed for each female to be mated. The mating period was then followed by an “egg laying deprivation period” according to the methods of Yang et al. During the deprivation period, 5 mated females were placed in a vial containing a moistened Whatman filter paper disc and yeast paste for a period of 24 hours (+/−3 hours). It should be noted that in our hands, this procedure did not effectively prevent the female flies from laying eggs, and we observed that they readily accepted the yeast paste as an egg laying substrate. This is consistent with the common practice of *Drosophila* researchers, where yeast paste is used in egg collections. Yeast paste is a well-known stimulant of egg laying in *Drosophila*. Three of the females were then used for oviposition choice experiments as described below.

### Preparation of media

3% agar medium was prepared by dissolving agar in double distilled H_2_O with liquid cycle autoclave and 1% agarose medium (with tastant) was prepared by dissolving agarose (and relevant tastant) in double distilled H_2_O using a microwave oven. The molten agar or agarose solution was cooled to approximately 50°C and Ethanol was added to a 1% final concentration.

### Egg Laying Assays

Preference indices were calculated using the formula: (number of eggs on sugar medium-number of eggs on non-sugar medium)/(total number of eggs). Trials in which females laid fewer than 5 eggs were excluded from the analyses.

Relative egg density in Figure 3 was calculated as follows: The plate was first divided into zones 1–7 (as shown in Figure 3) where the sucrose island was in zone 1. The center of the sucrose island was located 10 mm off center of the 50 mm chamber. This position of the island was chosen to mimic the position of the sucrose islands in the experimental setup shown in Figure 2D. The radius of the sucrose island was 3.75 mm. From the center of the sucrose zone, the radii of the concentric circles delineating each zone were 10 mm, 15 mm, 20 mm, 25 mm and 30 mm respectively. The areas of the zones (a_y_) were a_1_ = 44.2 mm^2^, a_2_ = 270.0 mm^2^, a_3_ = 392.7 mm^2^, a_4_ = 398.1 mm^2^, a_5_ = 361.9 mm^2^, a_6_ = 310.3 mm^2^, a_7_ = 186.4 mm^2^. The total number of eggs (n_xy_) laid in trial x was computed from the sum of the eggs in all zones (n_x_ = n_x1_+n_x2_+n_x3_+n_x4_+n_x5_+n_x6_+n_x7_). The percentage of eggs laid in each zone on a given trial (p_xy_ = n_xy_/n_x_) was used to calculate the average percentage of eggs laid across trials (p_y_ = (p_1y_+p_2y_+…+p_xy_)/x). The egg density (d_y_) in each zone was computed by dividing the percentage of eggs in the zone by the surface area for that zone (p_y_/a_y_). Finally, the relative egg density for each zone was determined by dividing the egg density of each zone by the total egg density (d_y_/(d_1_+d_2_+d_3_+d_4_+d_5_+d_6_+d_7_)).

Egg laying assays were performed in 19 mm caps from 15 ml Falcon tubes, or Falcon 35 or 60 mm (actual diameter is 50 mm) Petri dishes. In the 19 mm or 35 mm dishes, the dish was first supplied with1 ml or 5 ml molten 3% agar (1% EtOH) which was allowed to cool and solidify prior to the addition of 1% agarose egg laying substrates. We then pipetted 100 µL (for 19 mm chambers) or 200 µL (for 35 mm chambers) of molten 1% agarose plus 1%EtOH or 1% agarose plus EtOH containing the relevant tastant (Sucrose (Mallinckrodt), Fructose (Sigma), Glucose (Sigma), Caffeine (Sigma), NaCl (Mallinckrodt)) on either side of the chamber. Care was used to ensure that the shape and surface area of the two egg laying substrates were similar. For trials in which we sought to eliminate diffusion in the 19 mm chambers, no 3% agar was applied. Instead, 400 µL of molten 1% agarose plus 1%EtOH and 1% agarose plus EtOH containing the sucrose (Mallinckrodt)) were applied to opposite sides of the chamber.

In the 50 mm dishes, egg laying islands were prepared by using the wide end of a p1000 pipette tip (7 mm in height) as a mold. In trials with two islands, the molds were placed near the center of a 50 mm Petri dish separated by a distance of 5 mm. One of the islands was filled with molten 1% plain agarose with 1% ethanol and the other was filled with 100 mM sucrose in 1% molten agarose with 1% ethanol. After the agarose in the two islands solidified, a 3% agarose solution was pipetted into the plate so that it surrounded the islands and reached the 7 mm height. In experiments which allowed for diffusion in the larger chambers the pipette tip mold was placed 10 mm from the center of a 50 mm Petri dish and the area outside of the mold was filled with a 1% plain agarose solution with 1% ethanol. Once the surrounding 1% agarose solution had sufficiently solidified, the mold was carefully removed and 1% agarose 1% ethanol solution with or without 100 mM sucrose was pipetted into the hole in order to match the height of the surrounding agarose.

A small hole was drilled in the lid of the Petri dish to allow for the placement of unanesthetized flies into the chamber. Flies were aspirated into the chambers through the hole and scotch tape was used to seal the hole after flies were added. Flies were typically added to the egg-laying chamber approximately 2 hours prior to lights off (7pm). Egg laying assays in these and other chambers were carried out at 25°C, 75% relative humidity, on a 12 hour light-dark cycle for a period of 24 hours. The assay was stopped by removal of flies and eggs were counted.

To prepare small egg laying chambers we utilized the caps of 15 ml polypropylene tubes. Approximately 1 mL of the 3% agar substrate was pipetted into inverted caps, allowed to solidify and 100 µL of the 1% agar egg laying substrates was then applied, with the alternate substrates on opposite sides. The chambers were sealed with parafilm to prevent evaporation. Unanesthetized flies were loaded into the chamber through a small hole that was drilled through the side in the center of the chamber.

### Larval Foraging Assays

For larval foraging assays 19 mm and 35 mm chambers were prepared (as described for egg-laying assays) and aged for 24 hours in order to establish a diffusion gradient. For each trial, three newly hatched Canton-S larvae were placed on the plain agarose side of the chamber using forceps and observed for 30 minutes to determine whether any larvae crossed the chamber midline towards the sucrose-containing substrate (or plain substrate in the control condition). For larvae that did cross the chamber midline, the time required to cross was recorded. Five trials were conducted for each of four conditions: 19 mm plates with one sucrose half and diffusion allowed, 19 mm plates with one sucrose half and diffusion not allowed, 19 mm plates with no sucrose and diffusion allowed, and 35 mm plates with one sucrose half and diffusion allowed.
